# Supplementary figures and images for: Relationship between triterpenoid anticancer drug resistance, autophagy, and caspase-1 in adult T-cell leukemia
Source: PeerJ. 2016 May 12;4:e2026. doi: 10.7717/peerj.2026 (PMC4868592; doi:10.7717/peerj.2026)

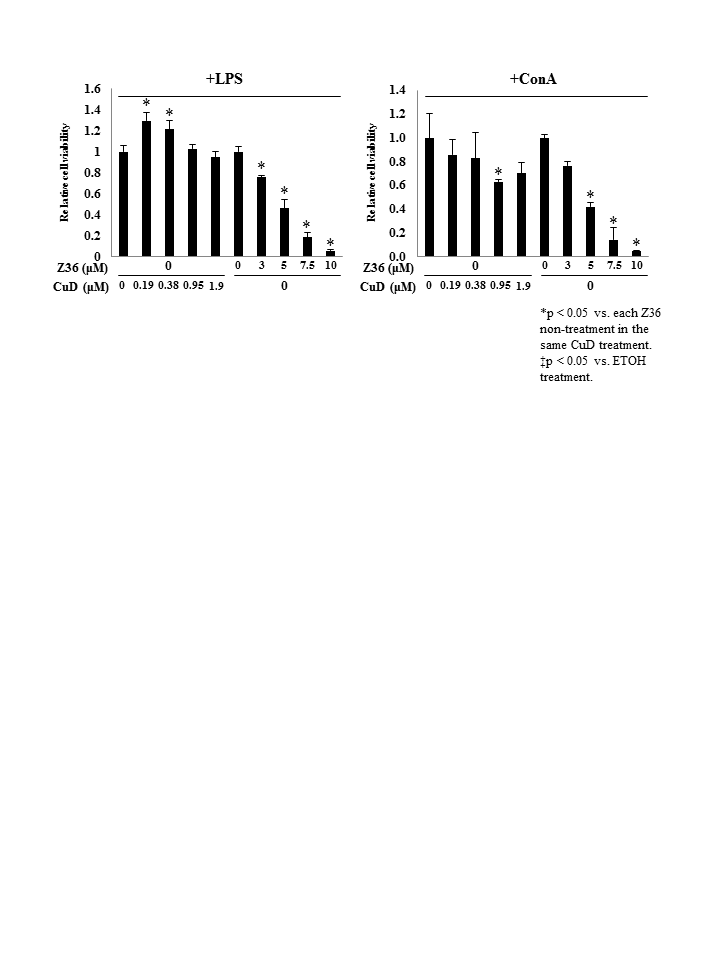

Supplement: Supplemental Information 1 — PBL from a heathy donor (1 × 10^4^) were treated with several concentrations of CuD or Z36, and stimulated with LPS (1 μg/ml; left panel) or ConA (5 μg/ml; right panel). [file peerj-04-2026-s001.png]

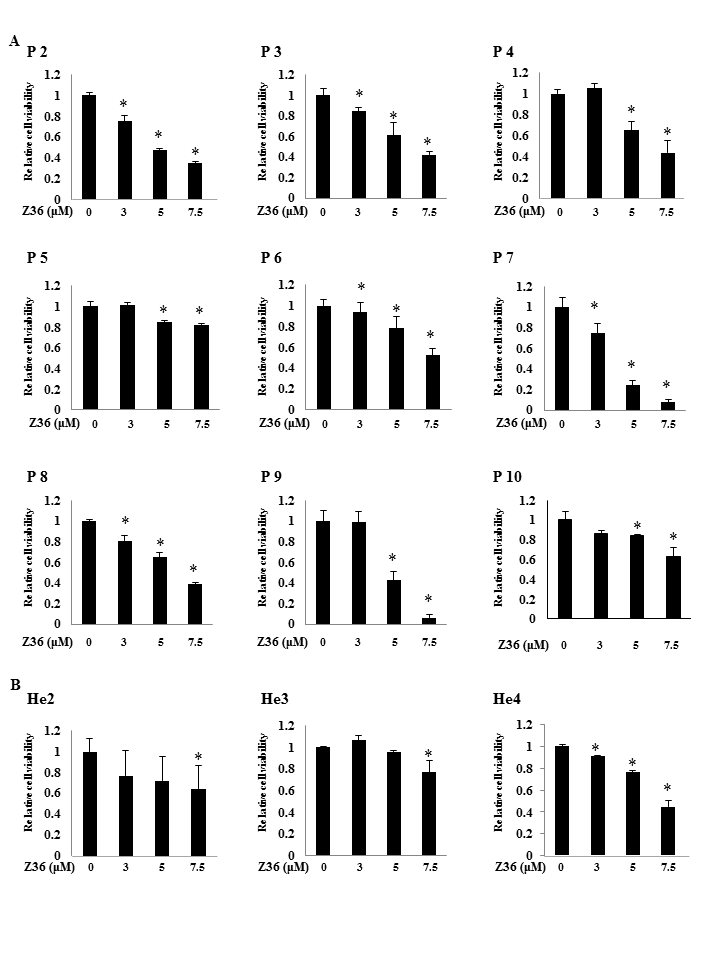

Supplement: Supplemental Information 2 — PBLs from ATL, T cell lymphoma patients, or healthy donors were treated with Z36 at the indicated concentrations for 24 h. The viability of cells was determined and expressed as the mean activity ± SD of quadruplicate wells. *P < 0.05 vs. vehicle. [file peerj-04-2026-s002.png]

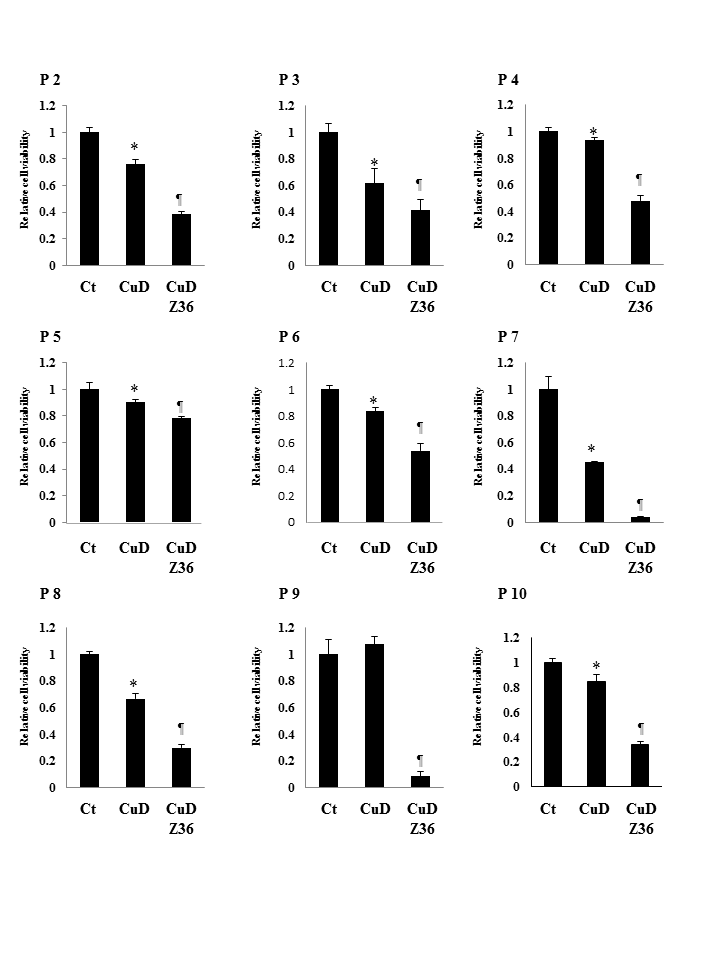

Supplement: Supplemental Information 3 — PBLs from ATL patients were treated with CuD (0.19 μM) and/or Z36 (7.5 μM) for 24 h. The viability of cells was determined and expressed as the mean activity ± SD of quadruplicate wells. *P < 0.05 vs. vehicle. [file peerj-04-2026-s003.png]

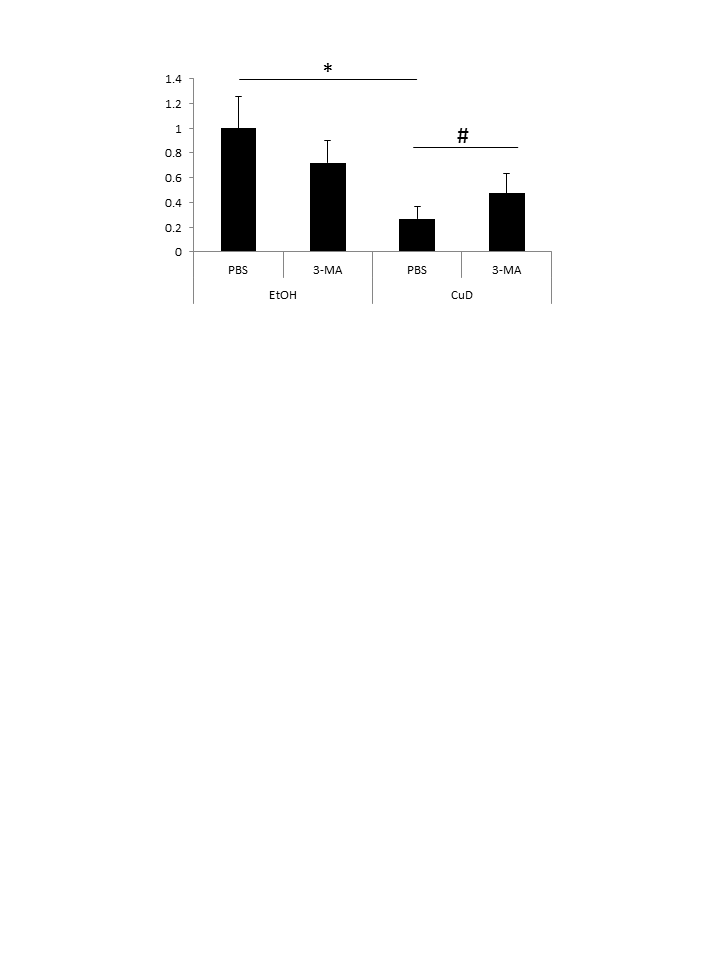

Supplement: Supplemental Information 4 — PBLs (1 × 10^4^/well) from ATL patients were treated with 3-MA (10 mM) or PBS for 24 h, and/or CuD (0.19 μM). *P < 0.05 vs. vehicle. Cell viability was determined using the Cell Titer- Glo assay. Results are expressed as mean count relative to controls and SD from quadruplicate cultures. *P < 0.05 vs. vehicle; #P < 0.05 vs. treatment with CuD only. [file peerj-04-2026-s004.png]
